# Supplementary figures and images for: Synthetic Amphipathic Helical Peptide L-37pA Ameliorates the Development of Acute Respiratory Distress Syndrome (ARDS) and ARDS-Induced Pulmonary Fibrosis in Mice
Source: Int J Mol Sci. 2024 Aug 1;25(15):8384. doi: 10.3390/ijms25158384 (PMC11312864; doi:10.3390/ijms25158384)

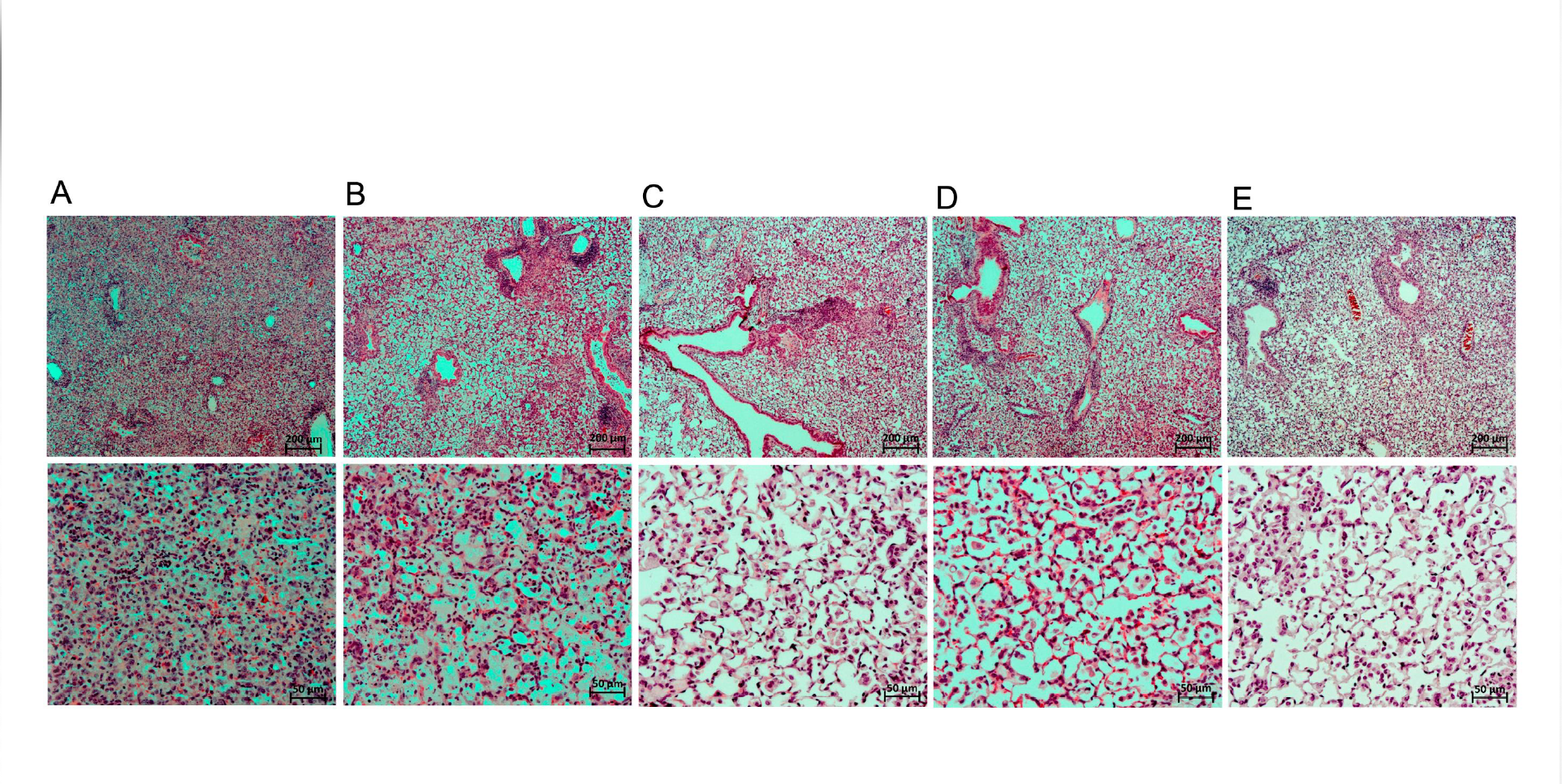

Supplement: Supplementary file 1 [file ijms-25-08384-s001.zip › Supplement Figure S1.tif]

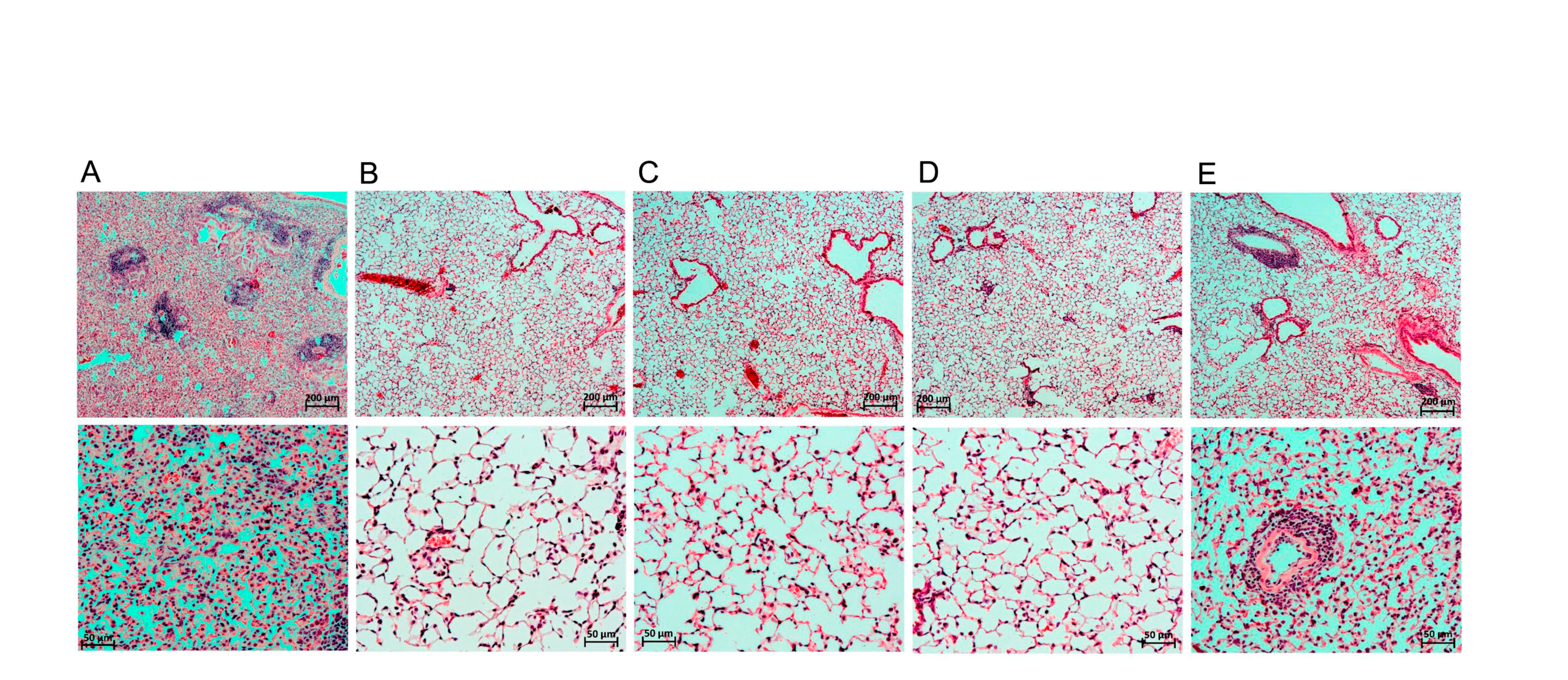

Supplement: Supplementary file 1 [file ijms-25-08384-s001.zip › Supplement Figure S2.tif]

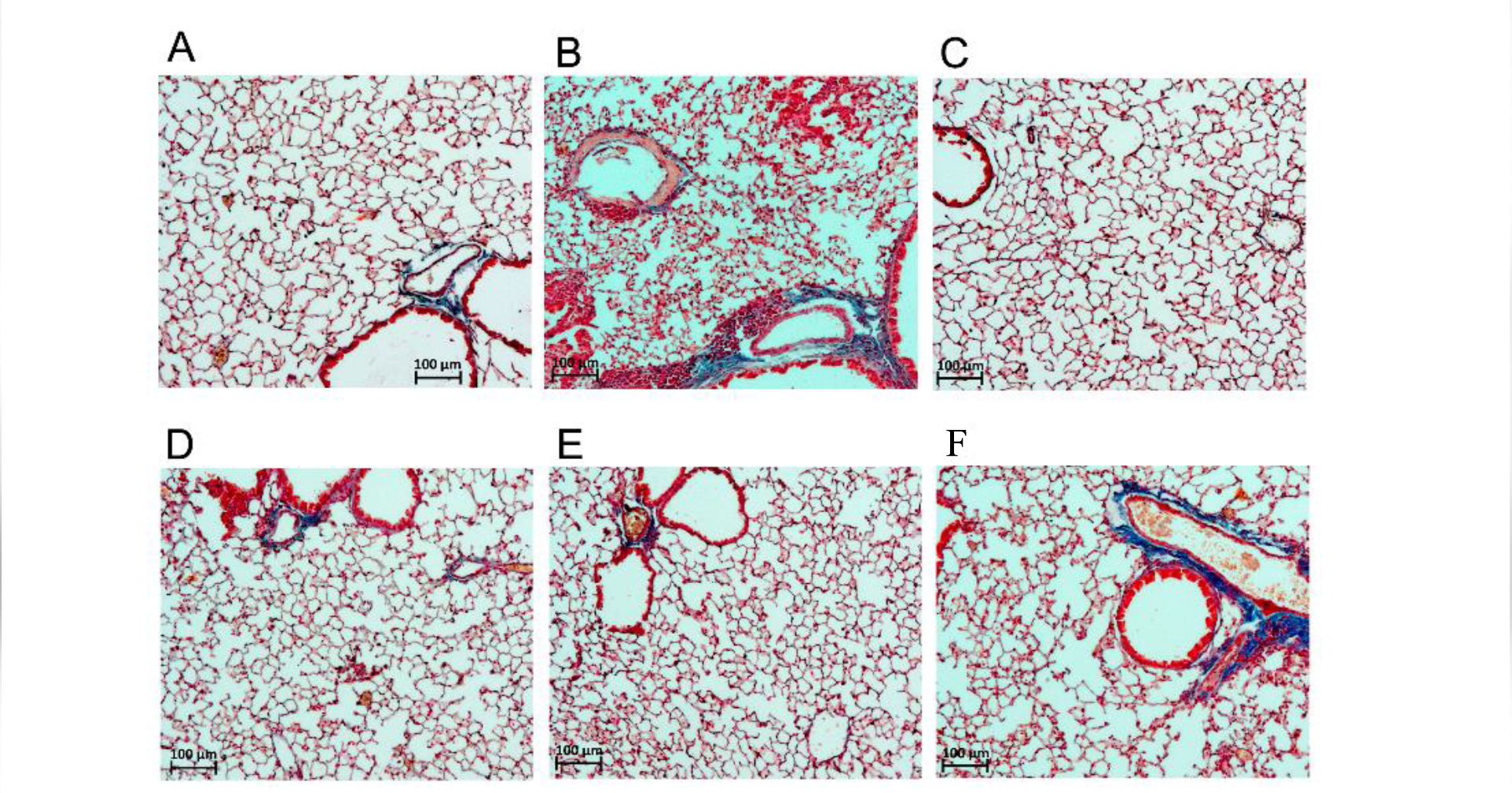

Supplement: Supplementary file 1 [file ijms-25-08384-s001.zip › Supplement Figure S3.tif]
